# Rat bone marrow mesenchymal stem cells (BMSCs) inhibit liver fibrosis by activating GSK3β and inhibiting the Wnt3a/β-catenin pathway

**DOI:** 10.1186/s13027-022-00432-4

**Published:** 2022-04-19

**Authors:** Zhaoguo Liu, Song Zhou, Ya Zhang, Ming Zhao

**Affiliations:** 1 The Second School of Clinical Medicine, Southern Medical University, Guangzhou, Guangdong Province China; 2 Liuzhou Worker’s Hospital, Liuzhou, Guangxi Province China; 3 Department of Organ Transplantation, The Second School of Clinical Medicine, Southern Medical University, Guangzhou, 510280 Guangdong Province China

**Keywords:** BMSCs, Liver fibrosis, Hepatic stellate cells, GSK3β, Wnt/β-catenin pathway

## Abstract

**Background:**

Bone marrow mesenchymal stem cells (BMSCs) can effectively alleviate liver fibrosis, which is a pathological injury caused by various chronic liver diseases. This study aimed to investigate the antifibrotic effects of BMSCs and elucidate the underlying mechanism by which BMSCs affect liver fibrosis in vitro and in vivo.

**Methods:**

After the rat liver fibrosis model was induced by continuous injection of carbon tetrachloride (CCl_4_), BMSCs were administered for 4 weeks, and histopathological analysis and liver function tests were performed. T6 hepatic stellate cells (HSC-T6 cells) were stimulated by TGF-β1, and the activation and proliferation of cells were analyzed by CCK-8 assays, flow cytometry, real-time PCR, western blotting and enzyme-linked immunosorbent assay (ELISA).

**Results:**

Our data demonstrated that BMSCs effectively reduced the accumulation of collagen, enhanced liver functionality and ameliorated liver fibrosis in vivo. BMSCs increased the sub-G1 population in HSC-T6 cells. In addition, coculture with BMSCs reduced the expression of α-SMA, collagen I, cyclin-D1, and c-Myc in HSC-T6 cells and activated the phosphorylation of GSK3β. The GSK3β inhibitor SB216763 reversed the effect of BMSCs. The Wnt/β-catenin signalling pathway was involved in BMSC-mediated inhibition of HSC-T6 cell activation.

**Conclusions:**

Our data suggested that BMSCs exerted antifibrotic effects by activating the expression of GSK3β and inhibiting the Wnt3a/β-catenin signalling pathway.

## Background

Although the liver is remarkably regenerative, fibrosis due to various chronic liver diseases can cause cirrhosis, hepatocellular carcinoma and liver failure [1, 2]. At present, few drugs can reverse or treat liver fibrosis, and the drugs have certain toxicities and adverse effects; therefore, drugs with curative effects and few side effects are urgently needed [3]. Currently, liver transplantation is considered to be an effective treatment option. However, the use of liver transplantation is restricted due to organ donor shortages, requirements for lifelong immunosuppression and surgical complications. Therefore, finding an alternative therapeutic strategy for treating liver fibrosis is urgently needed.

In recent years, there has been hope that stem cells can ameliorate hepatic disease due to advances in stem cell research [4, 5]. Bone marrow mesenchymal stem cells (BMSCs) have been extensively studied, and BMSCs can regulate nutritional factors and indirectly inhibit apoptosis. In addition, it was also found that BMSCs had tantifibrotic and angiogenic effects and played an important role in tissue repair [4, 6]. BMSCs have significant therapeutic potential in liver fibrosis, and it was reported that BMSCs could decrease hepatic fibrosis, improve hepatic function and contribute to hepatic regeneration [7, 8]. In addition, it has been reported that BMSCs can promote hepatocyte proliferation by inhibiting the activation of hepatic stellate cells and hepatocyte apoptosis [9]. In this study, we focused on the underlying mechanisms of liver fibrosis improvement.

An increasing number of molecules have been shown to play important roles in the initiation and development of fibrosis, and GSK3β is one of the important targets. GSK-3β, a key kinase in the Wnt/β-catenin pathway, participates in embryonic development, apoptosis, cell differentiation, translation and transcription [10, 11]. Activation of the Wnt/β-catenin pathway has been proven to be relevant to fibrosis of the lung, kidney, skin and liver [12, 13]. Previous studies have shown that Wnt/β-catenin signalling is one of the key pathways and is abnormally activated in many kinds of lung fibrosis, and the Wnt/β-catenin signalling pathway can inhibit the progression of pulmonary fibrosis when inhibitors are used [14]. Additionally, an inhibitor of CBP/β-catenin could exert an antifibrotic effect in vivo by inhibiting the activation of hepatic stellate cells (HSCs) and promoting inflammation resolution [15]. It was reported that the Wnt signalling pathway was upregulated in activated HSCs, and an inhibitor of GSK3β restrained the expression of α-SMA [16]. Moreover, BMSCs have been reported to attenuate the Wnt/β-catenin signalling pathway to relieve renal fibrosis and pulmonary fibrosis [17, 18].

In this study, we aimed to investigate the antifibrotic role of BMSCs and expose the underlying mechanism of BMSCs on liver fibrosis in vitro and in vivo. First, the rat liver fibrosis model was induced by the continuous injection of CCl4, and BMSCs were administered for 4 weeks. Second, histopathological analysis and liver function tests were performed in vivo. Finally, the activation and proliferation of HSC-T6 cells stimulated with TGF-β1 were analysed by CCK-8, flow cytometry, real-time PCR, western blotting and enzyme-linked immunosorbent assay (ELISA). This study was the first to validate the reduction in BMSCs in rat liver fibrosis and found that BMSC treatment exerted antifibrotic effects by activating the expression of GSK3β and inhibiting the Wnt3a/β-catenin signalling pathway. These findings may reveal strategies for the application of BMSCs as an effective to treat liver fibrosis.

## Materials and methods

### Cell culture

Rat BMSCs were purchased from Cyagen Biosciences(China). T6 hepatic stellate cells (HSC-T6 cells) were purchased from Procell Life Technology (China). BMSCs were cultured in OriCell Sprague Dawley Rat Bone Marrow Mesenchymal Stem Cell Basal Medium (Cyagen, Guangzhou, China), and HSC-T6 cells were cultured in Dulbecco’s modified Eagle’s medium (DMEM) (Gibco, Grand Island, USA) with 10% foetal bovine serum (FBS) (Gibco, New Zealand) and 1% penicillin streptomycin (Gibco, Grand Island, USA) at 37 °C and 5% CO_2_.

### CCl_4_-induced liver fibrosis in rats

Sprague Dawley (SD) rats (8 weeks old, female) were purchased from Guangdong Medical Experimental Animal Centre and were housed in a specific pathogen-free environment with a day and night cycle of 12 h. To induce the liver fibrosis model, rats were intraperitoneally (i.p.) injected with 30% carbon tetrachloride (CCl_4_) (MACKELIN, Shanghai, China) at a dose of 3 mL/kg body weight twice per week for 8 weeks. Then, CCl_4_-treated rats were randomly divided into two groups (n = 10): CCl_4_ + PBS (model group) and CCl_4_ + BMSCs (treatment group). Rats that were not treated with CCl_4_ served as the control group (n = 10). Rats in the CCl_4_ + BMSC group were injected with 1 × 10^6^/200 µL BMSCs through the tail vein, and rats in the CCl_4_ + phosphate buffer saline (PBS) group rats were injected with 200 µL PBS. Four weeks later, the rats were euthanized, and the serum and livers were collected to evaluate the therapeutic effects of BMSCs on liver fibrosis by western blotting and ELISA. The animal experiment protocols were approved by the Animal Experiment Ethics Committee of Southern Medical University (Ethics No.: L2017062).

### Cell coculture

Twenty-four-well plates with a Transwell chamber (0.4 μm pore size, Corning, USA) were used to physically separate the two cell populations. HSC-T6 cells were seeded in the lower chamber, and BMSCs were seeded in the upper chambe in DMEM with 10% FBS. After 24 and 48 h, the samples were collected for subsequent tests.

### Enzyme-linked immunosorbent assay (ELISA)

Blood samples and urine were collected from the rats, and serum was collected after centrifugation for 10 min at 3000 rpm. Aspartate aminotransferase (AST), alkaline phosphatase (ALP), *Helicobacter pylori* (HP) and catalase (CAT) levels in rat serumwere measured by ELISA kits (CUSCBIO, Wuhan, China). The concentrations of AST, ALP, HP and CAT were determined quantitatively according to the corresponding standard curves.

### mRNA extraction and real-time PCR

Total RNA was isolated from HSC-T6 cells according to the specifications of RNA the reagent (Invitrogen, California, USA), and reverse transcription of RNAs was performed by using the PrimeScript™ RT Master Mix kit (Takara Bio, Japan). Then, real-time PCR was conducted by using SYBR Premix Ex Tap™ II (TaKaRa, Tokyo, Japan). After normalization to glyceraldehyde-3-phosphate dehydrogenase (GAPDH), the relative amount of the PCR products was calculated using the 2 − ΔΔCt method. The primers were designed by Primer 5.0 and are shown in Table 1.

**Table 1:** The primer sequences used for RT-qPCR

Gene	Primer	ID
α-SMA	Forward: ACCATCGGGAATGAACGCTT Reverse: CTGTCAGCAATGCCTGGGTA	58
Collagen I	Forward: GAGACAGGCGAACAAGGTGA Reverse: GGGAGACCGTTGAGTCCATC	1277
Wnt3a	Forward: CGGGTTCTTCTCTGGTCCTTG Reverse: GGGCATGATCTCCACGTAGT	89,780
GAPDH	Forward: GCAAGTTCAACGGCACAG Reverse: GCCAGTAGACTCCACGACAT	2597

### Western blotting

HSC-T6 cells and liver tissue were obtained and lysed using radioimmunoprecipitation assay (RIPA) buffer (DGCS Biotechnology, China). The protein concentrations of the samples were measured using a BCA protein assay kit (Beyotime, Jiangsu, China). Then, the cell extracts were separated by 8% sodium dodecyl sulfate–polyacrylamide gel electrophoresis (SDS–PAGE) and transferred to a polyvinylidene fluoride (PVDF) membranes (Millipore, USA). Then, the samples were visualized by incubation with primary antibodie against alph-smooth muscle actin (α-SMA) (ab124964, Abcam, USA), collagen I (ab270993, Abcam, USA), matrix metalloproteinase-7 (MMP7) (3801T, CST, USA), Wnt3a (DF6113, Affinity, China), β-catenin (ab68183, Abcam, USA), p-GSK3β (AF2016, Affinity, China), cyclin-D1 (55506T, CST, USA), c-Myc (18583s, CST, USA) and GAPDH (AF0006, Beyotime, China) at 4 °C overnight, followed by incubation with HRP-labelled goat anti-mouse/rabbit IgG (H + L) secondary antibodies (A0216/A0208, Beyotime, China) for 1 h. The immune complexes were immunoblotted, and the immunodetection was performed by using enhanced chemiluminescence reagents ((FdBio, China).

### Cell counting kit-8(CCK-8) assay

A CCK-8 kit (Dojindo, Kyushu, Japan) was used to determine the proliferation of HSC-T6 cells. First, 5 × 10^3^ cells were seeded into 96-well plates CCK-8 reagent was added, and the plates were incubated for 2 h at 37 °C according to the manufacturer’s protocol. Optical densities were measured using a multifunctional microplate reader at 450 nm (OD450).

### Cell cycle assay

HSC-T6 cells were harvested and fixed for the cell cycle assay. The samples were fixed in 70% ethanol overnight at 4 ℃. After being centrifuged, the cells were stained with propidium iodide (PI) at 37 ℃ for 30 min using a Cell Cycle Analysis Kit (Beyotime, Jiangsu, China). The distribution of cells in the different phases of the cell cycle were analysed on a FACSCalibur flow cytometer (Becton Dickinson, New Jersey USA).

### Statistical analysis

The data are presented as the mean ± SD. All statistical analyses were performed by GraphPad software (v6.0). Statistical significance was determined using one-way analysis of variance (ANOVA) and Tukey’s test or Student’s t test. Allexperiments were repeated three times. Values of *p < 0.05, **p < 0.01, and ***p < 0.001 were considered significant.

## Results

### BMSCs alleviate liver fibrosis in CCl_4_-induced rats

**Fig. 1:**
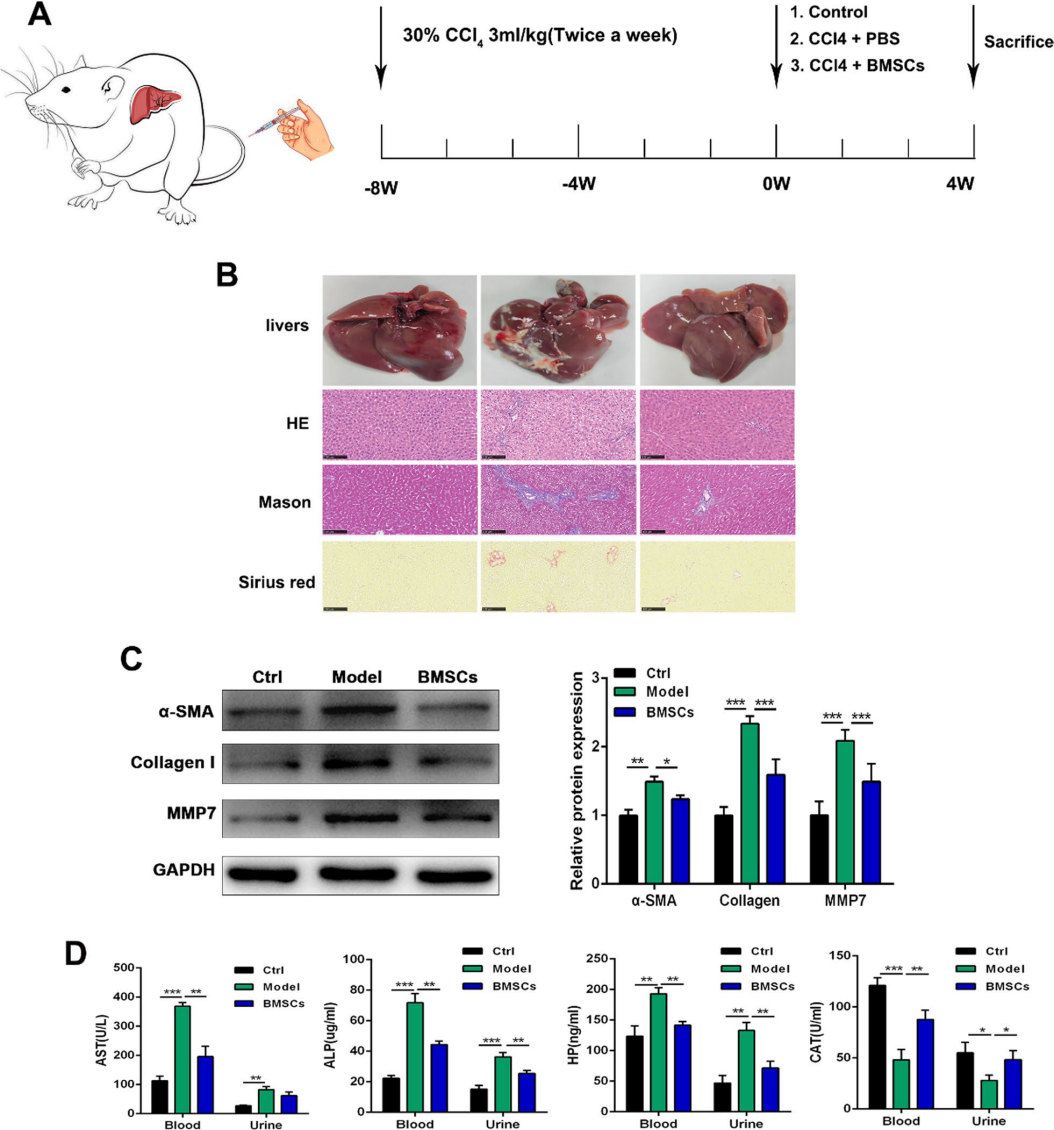
BMSCs alleviates liver fibrosis in CCl_4_ rats. **A** Schematic illustration showing the process of in vivo experiments. **B** The representative images showing the gross morphology of liver and the representative photomicrographs of liver tissue sections stained with H&E, Masson and Sirius Red (100 μm). **C** The western blotting analysis measuring α-SMA, Collagen I and MMP7 in liver fibrosis tissue. **D** The AST, ALP, HP and CAT levels of serum and urine by ELISA assay. Data were presented as the mean ± SD obtained from three separate experiments. *p < 0.05, **p < 0.01, ***p < 0.001

In this study, we used a CCl_4_-induced liver fibrosis model in rats to determine whether BMSCs could exert therapeutic effects on liver fibrosis. The rats were injected with CCl_4_ for 8 weeks and then treated with BMSCs for 4 weeks (Fig. 1A). The results showed that the livers in the model group were enlarged and coarse; however, control rat livers were smooth, had a uniform surface and had a soft texture; treated with BMSCs showed reductions in surface coarseness, and the livers were reddish, smoother and more lustrous than those in the model group (Fig. 1B). HE, Sirius red and Masson staining were used to examine liver fibrosis. Collagen deposition was markedly decreased in injured liver tissues from the BMSC-treated rats with hepatic fibrosis compared with those in model group. Western blotting showed that BMSCs could significantly inhibit the protein expression of α-SMA, collagen I, and MMP7 in fibrotic liver tissues (p < 0.05 or *p* < 0.001) (Fig. 1C). Moreover, the biochemical analyses were performed to evaluate the restoration of liver function after BMSC treatment. The results showed that serum and urine levels of AST, ALP and HP were significantly inhibited in the BMSCs group compared with the model group (*p* < 0.05 or *p* < 0.01). In addition, the serum and urine levelsl of CAT in the BMSC-treated group were higher than those in the model group (*p* < 0.05 or *p* < 0.01) (Fig. 1D). These results suggested that BMSC treatment could enhance liver function.

### BMSCs suppress the activation of TGF-β1-induced HSC-T6 cells

**Fig. 2:**
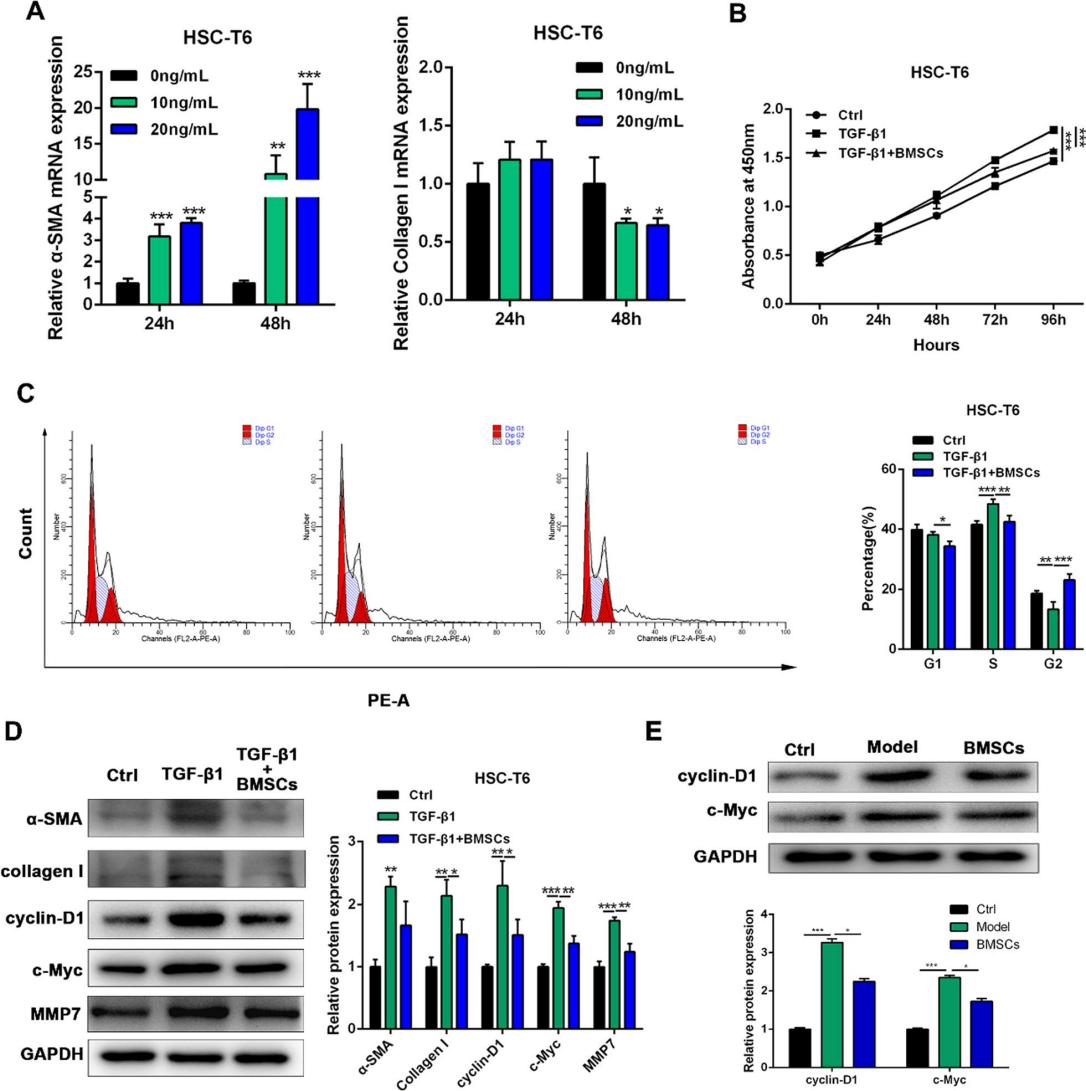
BMSCs suppresses TGF-β1-induced HSC-T6 activation. **A** The mRNA expression of α-SMA and Collagen I in HSC-T6 cells were detected by RT-qPCR after treatment with TGF-β1. **B** The cell viability of HSC-T6 cells was measured by CCK-8 analysis. **C** The cell cycle was detected by flow cytometry analysis of HSC-T6 cells in each group. **D** The protein expression of α-SMA, Col1α1, cyclin-D1, c-myc, and MMP7 from HSC-T6 cells was detected by western blotting. **E** The protein expression of cyclin-D1and c-myc from rat liver tissues was detected by western blotting. Data were presented as the mean ± SD obtained from three separate experiments. *p < 0.05, **p < 0.01, ***p < 0.001

TGF-β1 can activate hepatic stellate cells and promote liver fibrosis. In this study, 10 ng/ml and 20 ng/ml TGF-β1 significantly increased the mRNA expression of α-SMA, and collagen I (Fig. 2A). To investigate the inhibitory effect of BMSCs on stellate cells, a coculture assay was used to determine the effects of BMSCs on the proliferation of stellate cells. The results showed that (Fig. 2B, C) TGF-β1 significantly increased HSC-T6 cell proliferation, while coculture with BMSCs inhibited HSC-T6 proliferation as shown by CCK-8 and cell cycle assays. Compared to the TGF-β1 group, the coculture group had significantly inhibited protein expression of α-SMA, collagen I, cyclin-D1, c-Myc and MMP7 (*p* < 0.01 or *p* < 0.001) (Fig. 2D). BMSCs also significantly inhibited the protein expression of cyclin D1 and c-Myc in liver tissue, as shown by western blotting (*p* < 0.05) (Fig. 2E).

### The effects of the Wnt/β-catenin signalling pathway on the proliferation and activation of HSC-T6 cells

**Fig. 3:**
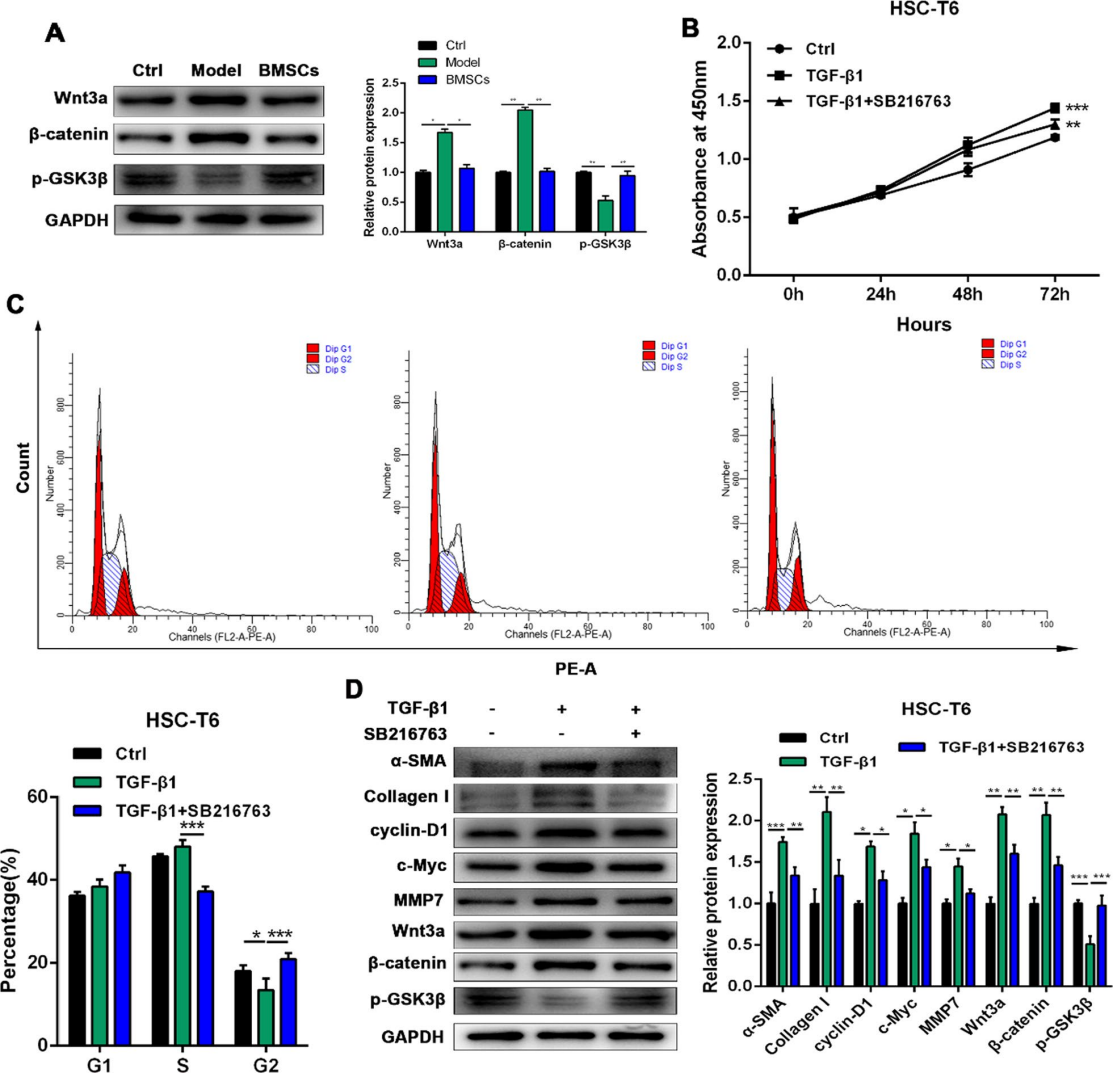
Effects of Wnt/β-catenin signaling pathway on proliferation and activation of HSC-T6 cells. **A** The protein expression of Wnt3a, β-catenin and p-GSK3β from rat liver tissues was detected by western blotting. **B** The cell viability of HSC-T6 cells was measured by CCK-8 analysis. **C** The cell cycle was detected by flow cytometry analysis of HSC-T6 cellsp. **D** The protein expression of α-SMA, Collagen I, cyclin-D1, c-Myc, MMP7, Wnt3a, β-catenin and p-GSK3β in HSC-T6 cells was detected by western blotting. Data were presented as the mean ± SD obtained from three separate experiments. *p < 0.05, **p < 0.01, ***p < 0.001

The expression levels of proteins involved in the Wnt/β-catenin signalling pathway in rat liver tissues were analysed by western blotting (Fig. 3A). Compared to that in the model group, the protein expression of Wnt3a (*p* < 0.05) and β-catenin (*p* < 0.01) was significantly inhibited in the BMSC treatment group, while the expression of p-GSK3β in the BMSC treatment group was significantly increased (*p* < 0.01), which suggested that BMSCs could effectively activate GSK3β and inhibit the Wnt/β-catenin signalling pathway in rat liver fibrosis. CCK-8 and cell cycle assays showed that TGF-β1 significantly increased HSC-T6 proliferation, while FH535, a specific inhibitor of Wnt/β-catenin signalling, inhibited the proliferation of HSC-T6 cells (Fig. 3B, C). As shown in Fig. 3D, FH535 blocked the ability of TGF-β1 to increase the protein expression of α-SMA (*p* < 0.01), collagen I (*p* < 0.01), cyclin-D1 (*p* < 0.05), c-Myc (*p* < 0.05), MMP7 (*p* < 0.05), Wnt3a (*p* < 0.01) and β-catenin (*p* < 0.01) in the HSC-T6 cells. FH535 also promoted GSK3β phosphorylation (*p* < 0.001). These results suggested that the Wnt/β-catenin signalling pathway might be involved in the activation of HSC-T6 cells and liver fibrosis.

### BMSCs effectively inhibit liver fibrosis via the Wnt3/β-catenin signalling pathway in HSC-T6 cells

**Fig. 4:**
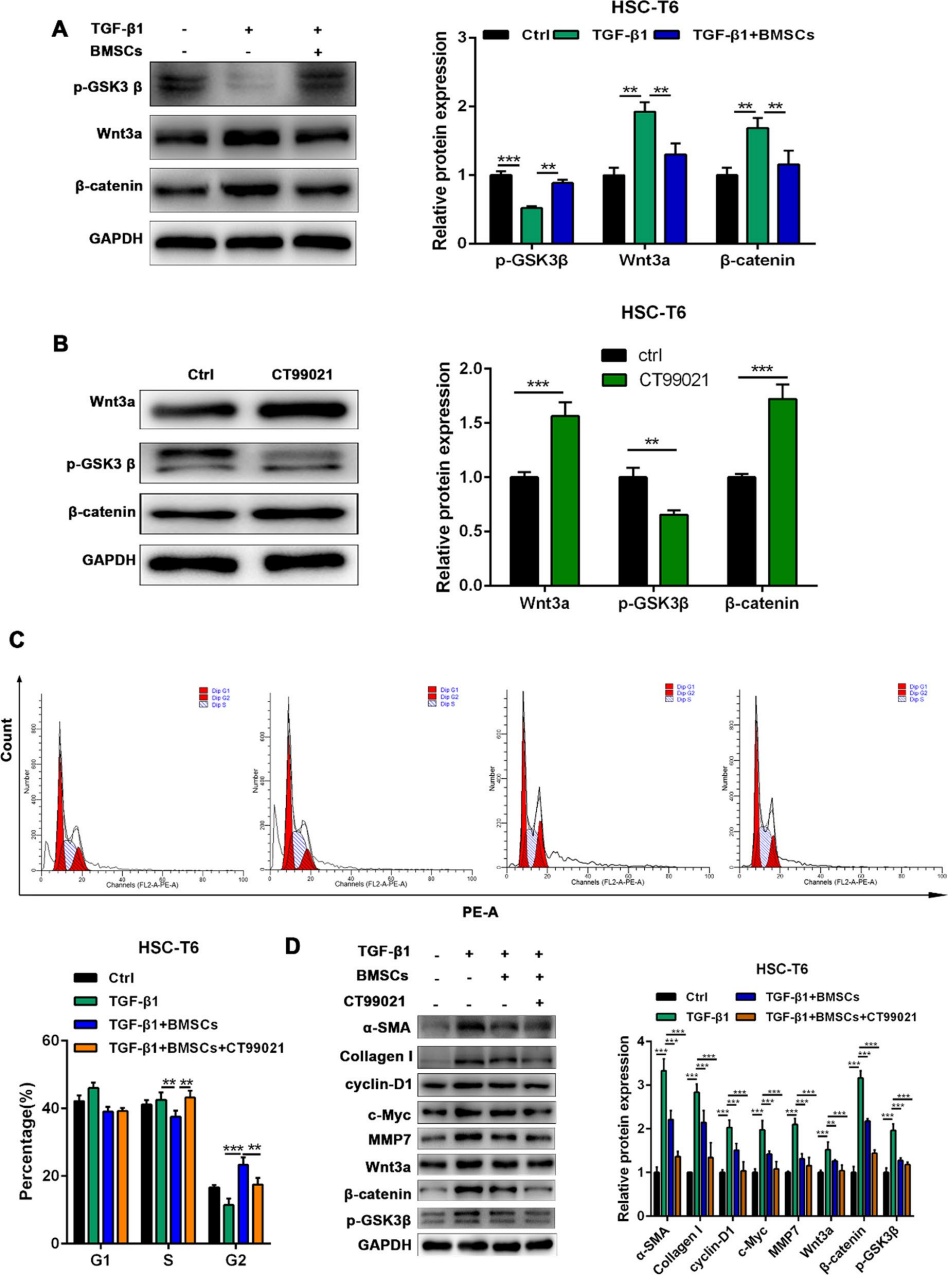
BMSCs effectively inhibits liver fibrosis through the Wnt3/β-catenin signaling in HSC-T6 cells. **A** The protein expression of Wnt3a, β-catenin and p-GSK3β in HSC-T6 cells detected by western blotting. **B** The protein expression of Wnt3a, β-catenin and p-GSK3β in HSC-T6 cells detected by western blotting assay after using CT99021. **C** The cell cycle was detected by flow cytometry analysis of HSC-T6 cells. **D** The protein expression of α-SMA, Collagen I, cyclin-D1, c-Myc, MMP7, Wnt3a, β-catenin and p-GSK3β in HSC-T6 cells was detected by western blotting in each group. Data were presented as the mean ± SD obtained from three separate experiments. *p < 0.05, **p < 0.01, ***p < 0.001

TGF-β1 significantly increased the protein expression of Wnt3a (*p* < 0.01) and β-catenin (*p* < 0.01) and inhibited GSK3β phosphorylation (*p* < 0.001). BMSC coculture attenuated the effect of TGF-β1 on HSC-T6 cells (*p* < 0.01) (Fig. 4A). To analyse the relationship between BMSCs and the Wnt/β-catenin signalling pathway in TGF-β1-induced activation of HSC-T6 cells in vitro, CT99021, an activator of the Wnt/β-catenin signalling pathway, was added to HSC-T6 cells. The results showed that CT99021 could significantly promote the expression of Wnt3a (*p* < 0.001) and β-catenin (*p* < 0.001), and it could also inhibit the phosphorylation of GSK3β (*p* < 0.01) (Fig. 4B). Coculture with BMSCs attenuated TGF-β1-mediated promotion of HSC-T6 cell proliferation; however, the addition of CT99021 abrogated the effects of BMSCs (Fig. 4C). In addition, CT99021 reversed the inhibitory effect of BMSCs on the proliferation and activation of HSC-T6 cells and promoted the expression of α-SMA, cyclin-D1, c-Myc, collagen I, MMP7, Wnt3a and β-catenin in the HSC-T6 cells (Fig. 4D). Therefore, BMSCs might effectively inhibit liver fibrosis through Wnt3/β-catenin signalling in HSC-T6 cells.

## Discussion

Almost all chronic liver injury causes liver fibrosis, but the current clinical treatment of liver fibrosis is not satisfactory, and there is a lack of effective drugs, resulting in the development of cirrhosis or hepatocellular carcinoma in some patients and leading to more than 1 million deaths every year [19, 20]. Chronic liver fibrosis greatly threatens human health, and its mechanism is complex due to an imbalance in the synthesis and degradation of extracellular matrix (EMC) during liver injury, which leads to the pathological deposition of fibrous connective tissue in liver cells [21, 22]. Liver fibrosis can be reversed to a certain extent, and the key to reversing liver fibrosis is early and accurate diagnosis and treatment. Therefore, finding an alternative therapeutic strategy for treating liver fibrosis is urgently needed. In our study, we investigated the antifibrotic role of BMSCs and elucidated the underlying mechanism of BMSCs on liver fibrosis in vitro and in vivo. This study was the first to validate the reduction in BMSCs in rat liver fibrosis and found that BMSC treatment exerted antifibrotic effects by activating the expression of GSK3β and inhibiting the Wnt3a/β-catenin signalling pathway.

To investigate the therapeutic effects of BMSCs on the CCl_4_-induced rat liver fibrosis model histopathological analysis and liver function tests were performed in vivo. The data revealed that BMSC treatment could reduce the protein expression of α-SMA, collagen I, and MMP7, and it could correct the CCl_4_-induces increases in the expression of AST, ALT and HP. Moreover, BMSCs also reduced the rat liver tissue fibrosis index, decreased fatty degeneration and significantly enhanced liver functionality. These results suggested that BMSC treatment had an effect on the rat liver fibrosis model induced by CCl_4_.

Hepatic stellate cells (HSCs), which are the main effector cells in liver fibrosis, play an important role in liver fibrosis [23, 24]. HSCs are generally quiescent. During chronic liver injuries, HSCs can be activated to become contractile myofibroblast-like cells and lead to collagen accumulation [25, 26]. Inhibition of HSC activation and proliferation has been regarded as the primary approach to treat liver fibrosis [27]. TGF-β, an important regulatory factor in the development of liver fibrosis, is involved in the activation and proliferation of HSCs through the upregulation of excessive ECM deposition [28, 29]. Our study confirmed that TGF-β could increase the expression of α-SMA and collagen I increase the sub-G1 portion of cells and promote the activation and proliferation of HSC-T6 cells. More importantly, our study also showed that BMSCs inhibited the activation and proliferation of HSCs by decreasing the expression levels of α-SMA, collagen I and several proteins involved in proliferation.

In recent years, the Wnt/β-catenin signalling pathway has been confirmed to be involved in the pathogenesis of different kinds of diseases [30]. Some studies have demonstrated that the Wnt/β-catenin signalling pathway is activated in many types of human organ fibrosis [31, 32]. It has been reported that activation of the Wnt/β-catenin signalling pathway leads to enhanced activation of HSCs by TGF-β1 [33]. Moreover, the Wnt/β-catenin signalling pathway is abnormally activated in silica-induced pulmonary fibrosis, and BMSCs may attenuate rat pulmonary fibrosis by inhibiting its expression [18]. In this study, the protein expression of Wnt and β-catenin in tissues or cells was decreased when BMSCs were used to treat liver fibrosis, while the protein expression of P-GSK3β was increased. This result suggests that the Wnt/β-catenin signalling pathway plays an important role in BMSC treatment of liver fibrosis.

To further identify the role of the Wnt/β-catenin signalling pathway in treatment of liver fibrosis, CT99021 was added to HSC-T6 cells that were cocultured with BMSCs, and CT99021 could abrogate the effect of BMSCs on Wnt3/β-catenin signalling in HSC-T6 cells. CT99021 reversed the inhibitory effect of BMSCs on the proliferation and activation of HSC-T6 cells and promoted the expression of α-SMA, cyclin-D1, c-Myc, collagen I, MMP7, Wnt3a and β-catenin in HSC-T6 cells. These results suggest that BMSCs can effectively inhibit liver fibrosis through Wnt3/β-catenin signalling.

## Conclusions

In summary, our study showed that BMSCs could effectively alleviate the rat liver fibrosis and inhibit the activation and proliferation of HSC-T6 cells. These effects were associated with interference with the Wnt/β-catenin signalling pathway in hepatocytes.

## Data Availability

The datasets used and/or analyzed during the current study are available from the corresponding author on reasonable request.

## Abbreviations

BMSCs: Bone marrow mesenchymal stem cells
CCl_4_: Carbon tetrachloride
ELISA: Enzyme-linked immunosorbent assay

## References

[1] Friedman S (2003). Liver fibrosis—from bench to bedside. J Hepatol.

[2] Wynn T, Ramalingam T (2012). Mechanisms of fibrosis: therapeutic translation for fibrotic disease. Nat Med.

[3] Hu Z, You P, Xiong S, Gao J, Tang Y, Ye X, Xia Y, Zhang D, Liu Y (2017). Carapax Trionycis extracts inhibit fibrogenesis of activated hepatic stellate cells via TGF-β1/Smad and NFκB signaling. Biomed Pharmacother.

[4] Watanabe Y, Tsuchiya A, Seino S, Kawata Y, Kojima Y, Ikarashi S, Starkey Lewis P, Lu W, Kikuta J, Kawai H (2019). Mesenchymal stem cells and induced bone marrow-derived macrophages synergistically improve liver fibrosis in mice. Stem Cells Transl Med.

[5] Chen L, Zhang C, Chen L, Wang X, Xiang B, Wu X, Guo Y, Mou X, Yuan L, Chen B (2017). Human menstrual blood-derived stem cells ameliorate liver fibrosis in mice by targeting hepatic stellate cells via paracrine mediators. Stem Cells Transl Med.

[6] Lan L, Liu R, Qin L, Cheng P, Liu B, Zhang B, Ding S, Li X (2018). Transplantation of bone marrow-derived endothelial progenitor cells and hepatocyte stem cells from liver fibrosis rats ameliorates liver fibrosis. World J Gastroenterol.

[7] Oyagi S, Hirose M, Kojima M, Okuyama M, Kawase M, Nakamura T, Ohgushi H, Yagi K (2006). Therapeutic effect of transplanting HGF-treated bone marrow mesenchymal cells into CCl4-injured rats. J Hepatol.

[8] Huang B, Cheng X, Wang H, Huang W, la Ga Hu Z, Wang D, Zhang K, Zhang H, Xue Z, Da Y (2016). Mesenchymal stem cells and their secreted molecules predominantly ameliorate fulminant hepatic failure and chronic liver fibrosis in mice respectively. J Transl Med.

[9] El Baz H, Demerdash Z, Kamel M, Atta S, Salah F, Hassan S, Hammam O, Khalil H, Meshaal S, Raafat I (2018). Transplant of hepatocytes, undifferentiated mesenchymal stem cells, and in vitro hepatocyte-differentiated mesenchymal stem cells in a chronic liver failure experimental model: a comparative study. Exp Clin Transplant.

[10] Zheng H, Yang Z, Xin Z, Yang Y, Yu Y, Cui J, Liu H, Chen F (2020). Glycogen synthase kinase-3β: a promising candidate in the fight against fibrosis. Theranostics.

[11] Chen H, Gan Q, Yang C, Peng X, Qin J, Qiu S, Jiang Y, Tu S, He Y, Li S (2019). A novel role of glutathione S-transferase A3 in inhibiting hepatic stellate cell activation and rat hepatic fibrosis. J Transl Med.

[12] Edeling M, Ragi G, Huang S, Pavenstädt H, Susztak K (2016). Developmental signalling pathways in renal fibrosis: the roles of Notch, Wnt and Hedgehog. Nat Rev Nephrol.

[13] Monga S (2015). β-Catenin signaling and roles in liver homeostasis, injury, and tumorigenesis. Gastroenterology.

[14] Wang C, Dai J, Sun Z, Shi C, Cao H, Chen X, Gu S, Li Z, Qian W, Han X (2015). Targeted inhibition of disheveled PDZ domain via NSC668036 depresses fibrotic process. Exp Cell Res.

[15] Osawa Y, Oboki K, Imamura J, Kojika E, Hayashi Y, Hishima T, Saibara T, Shibasaki F, Kohara M, Kimura K (2015). Inhibition of cyclic adenosine monophosphate (cAMP)-response element-binding protein (CREB)-binding protein (CBP)/β-catenin reduces liver fibrosis in mice. EBioMedicine.

[16] Kordes C, Sawitza I, Häussinger D (2008). Canonical Wnt signaling maintains the quiescent stage of hepatic stellate cells. Biochem Biophys Res Commun.

[17] Zhang F, Wan X, Cao Y, Sun D, Cao C (2018). Klotho gene-modified BMSCs showed elevated antifibrotic effects by inhibiting the Wnt/β-catenin pathway in kidneys after acute injury. Cell Biol Int.

[18] Zhang E, Yang Y, Chen S, Peng C, Lavin M, Yeo A, Li C, Liu X, Guan Y, Du X (2018). Bone marrow mesenchymal stromal cells attenuate silica-induced pulmonary fibrosis potentially by attenuating Wnt/β-catenin signaling in rats. Stem Cell Res Ther.

[19] Tsochatzis E, Bosch J, Burroughs A (2014). Liver cirrhosis. Lancet.

[20] Schuppan D, Ashfaq-Khan M, Yang A, Kim Y (2018). Liver fibrosis: direct antifibrotic agents and targeted therapies. Matrix Biol.

[21] Geervliet E, Moreno S, Baiamonte L, Booijink R, Boye S, Wang P, Voit B, Lederer A, Appelhans D, Bansal R (2021). Matrix metalloproteinase-1 decorated polymersomes, a surface-active extracellular matrix therapeutic, potentiates collagen degradation and attenuates early liver fibrosis. J Control Release.

[22] Hussein K, Park K, Yu L, Kwak H, Woo H (2020). Decellularized hepatic extracellular matrix hydrogel attenuates hepatic stellate cell activation and liver fibrosis. Mater Sci Eng C Mater Biol Appl.

[23] Friedman S (2008). Mechanisms of hepatic fibrogenesis. Gastroenterology.

[24] Mederacke I, Hsu C, Troeger J, Huebener P, Mu X, Dapito D, Pradere J, Schwabe R (2013). Fate tracing reveals hepatic stellate cells as dominant contributors to liver fibrosis independent of its aetiology. Nat Commun.

[25] Brempelis K, Crispe I (2016). Infiltrating monocytes in liver injury and repair. Clin Transl Immunol.

[26] Tsuchida T, Friedman S (2017). Mechanisms of hepatic stellate cell activation. Nat Rev Gastroenterol Hepatol.

[27] Zhang C, Yuan W, He P, Lei J, Wang C (2016). Liver fibrosis and hepatic stellate cells: etiology, pathological hallmarks and therapeutic targets. World J Gastroenterol.

[28] Seki E, De Minicis S, Osterreicher C, Kluwe J, Osawa Y, Brenner D, Schwabe R (2007). TLR4 enhances TGF-beta signaling and hepatic fibrosis. Nat Med.

[29] Dooley S, Delvoux B, Streckert M, Bonzel L, Stopa M, ten Dijke P, Gressner A (2001). Transforming growth factor beta signal transduction in hepatic stellate cells via Smad2/3 phosphorylation, a pathway that is abrogated during in vitro progression to myofibroblasts. TGFbeta signal transduction during transdifferentiation of hepatic stellate cells. FEBS Lett.

[30] Zhou D, Wang X, Wang Y, Xiang X, Liang Z, Zhou Y, Xu A, Bi C, Zhang L (2016). MicroRNA-145 inhibits hepatic stellate cell activation and proliferation by targeting ZEB2 through Wnt/β-catenin pathway. Mol Immunol.

[31] Guo Y, Xiao L, Sun L, Liu F (2012). Wnt/beta-catenin signaling: a promising new target for fibrosis diseases. Physiol Res.

[32] Nishikawa K, Osawa Y, Kimura K (2018). Wnt/β-catenin signaling as a potential target for the treatment of liver cirrhosis using antifibrotic drugs. Int J Mol Sci.

[33] Chen Y, Chen X, Ji Y, Zhu S, Bu F, Du X, Meng X, Huang C, Li J (2020). PLK1 regulates hepatic stellate cell activation and liver fibrosis through Wnt/β-catenin signalling pathway. J Cell Mol Med.

